# The association between TNF-α 238A/G and 308A/G polymorphisms and juvenile idiopathic arthritis: An updated PRISMA-compliant meta-analysis

**DOI:** 10.1097/MD.0000000000012883

**Published:** 2018-10-26

**Authors:** Xing-yan Li, Chun-hua Liang, Virginia Parkman, Zheng-tao Lv

**Affiliations:** a Department of Orthopedics, The Third Affiliated Hospital of Guangxi Medical University, Nanning, Guangxi, China; b Harvard School of Dental Medicine, Division of Bone and Mineral Research, Department of Oral Medicine, Infection and Immunity, Boston, MA; c Department of Orthopedics, Tongji Hospital, Tongji Medical College, Huazhong University of Science and Technology, Wuhan, China.

**Keywords:** juvenile idiopathic arthritis, meta-analysis, polymorphism, TNF-α

## Abstract

**Objective:**

A previous meta-analysis concluded that TNF-α 238A/G and TNF-α 308A/G polymorphisms were not associated with the risk of juvenile idiopathic arthritis (JIA) in the overall population or Caucasian subjects. With the publication of a fair number of studies on the association between TNF-α polymorphisms and JIA in recent years, we conducted this updated meta-analysis to make a more accurate evaluation of such relationship.

**Methods:**

We adopted PubMed, EMBASE, ISI Web of Science and CNKI to identify observational studies that addressed the association between TNF-α polymorphisms and risk for JIA. The allelic effect of variant A for the risk of JIA was expressed as odds ratio (OR) along with the associated 95% confidence interval (95% CI). Meta-analyses were performed by pooling ORs and 95%CI from included studies using RevMan 5.3 software. The stratified-analysis based on ethnicity was performed to confirm the ethnicity-dependent effect on the relationship.

**Results:**

A total of 15 case-control studies including 2845 patients in JIA groups and 4771 patients in control groups were included in our study. The findings indicated a statistically significant association between the A allele of the TNF-alpha 238A/G polymorphism and the decreased JIA risk in Caucasians (*P* = .0002). The study in Iranian showed similar results (*P* = .0002) whereas the studies in other ethnicities failed to replicate this finding: Han (*P* = .29), Mexican (*P* = .64) and Turkish population (*P* = .32). TNF-α 308A/G was not statistically associated with JIA in overall subjects or Caucasians.

**Conclusion:**

Our study confirmed the protective role of the A allele in TNF-α 238A/G but not TNF-α 308A/G against the occurrence of JIA in the Caucasian population. To exactly validate the correlation between TNF-α polymorphisms and JIA in other ethnic backgrounds, additional studies are required.

## 1 Introduction

Juvenile idiopathic arthritis (JIA) is 1 of the most common childhood rheumatic diseases, which is characterized by arthritis for more than 6 weeks with onset before age of 16 years, and in severe cases may be debilitating, even life-threatening. According to the International League of Associations for Rheumatology (ILAR), JIA is a term which encompasses all forms of arthritis, including systemic arthritis, oligo-arthritis, polyarthritis rheumatoid factor (RF) negative, polyarthritis RF positive, psoriatic arthritis, enthesitis-related arthritis (ERA), and undifferentiated arthritis, but not a single disease.^[1,2]^ One of the most intriguing questions of JIA is what triggers the disease and decides its pathogenesis. Despite the heterogeneity of JIA, genetic susceptibility, balancing tolerance and inflammation are all considered to be closely related to the subtypes of JIA.^[3–5]^

TNF-α is a multifunctional pro-inflammatory cytokine that belongs to the TNF superfamily, which is mainly secreted by macrophages.^[6]^ This pro-inflammatory cytokine is involved in the regulation of a wide spectrum of biological processes including cell proliferation, differentiation, apoptosis, lipid metabolism, and coagulation, through binding to its receptors TNFRSF1A/TNFR1 and TNFRSF1B/TNFBR.^[7]^ As 1 of the members of the TNF superfamily, it has been implicated in a variety of pathological processes including autoimmune diseases,^[8]^ insulin resistance,^[9]^ inflammatory diseases^[10]^ and cancer.^[11]^ Currently, the combination of methotrexate and tumor necrosis factor α blockade is the primary treatment program for JIA,^[12]^ in spite of the exception of a possible small increased risk of lymphoma, other types of cancer and serious infections in children.^[13,14]^ Additionally, a large number of studies have confirmed elevated levels of TNF-α in the serum and synovial fluid of JIA patients, which indicates the potential relationship between elevated level of TNF-α and occurrence of JIA.^[15]^

Single nucleotide polymorphisms (SNPs) are single nucleotide variations that can occur at any site in the DNA, which distribute widely across the human genome and may influence transcription of protein. Despite lots of research that has been conducted to study the association of SNPs of TNF promoter −308A/G and −238A/G and JIA susceptibility, the outcomes have been inconsistent. As far as we know, only 1 meta-analysis has been carried out to assess the correlation between TNF-α polymorphisms and JIA, the authors concluded that there was no association between the A allele (the variant allele) of TNF-α 238A/G or TNF-α 308A/G and JIA in European populations.^[16]^ However, the sample size of that meta-analysis was not large enough, and they included an article that did not fit the inclusion criteria. Therefore, with the publication of a fair number of studies on the association between TNF-α polymorphisms and JIA in recent years, we conducted this updated meta-analysis to critically evaluate all the currently available evidence and to determine whether there is any association between SNPs of TNF-α promoter and risk of JIA.

## 2 Methods

This meta-analysis was performed in accordance with the PRISMA guideline.^[17]^ This study does not need an ethical approval because it is a meta-analysis and does not include any data directly linked to individual patient information.

### 2.1 Literature search strategy

A comprehensive literature search was performed in PubMed, EMBASE, ISI Web of Science, and CNKI to identify potentially eligible studies that were published before July 1, 2017. No language restriction was imposed. We used a structural literature search strategy to retrieve observational studies that analyzed the association between TNF-α polymorphisms and JIA, the literature search strategy used in English databases was as follow: (SNP or SNPs or “Polymorphism, Single Nucleotide”[Mesh]) and (“Arthritis, Juvenile”[Mesh] or juvenile arthritis or JIA or juvenile rheumatoid arthritis or juvenile chronic arthritis or JIA or JRA) and (“Tumor Necrosis Factor-α”[Mesh] or TNF-α or Tumor Necrosis Factor-α). In CNKI, we searched relevant Chinese publications using “TNF-α” and “Duo Tai Xing” and “Guan Jie Yan”. We used “Guan Jie Yan” which means “arthritis” to increase the sensitivity of our literature search in CNKI. Additionally, we screened the reference lists of relevant reviews and meta-analyses to avoid missing eligible studies.

### 2.2 Inclusion criteria and exclusion criteria

Available data were extracted from published articles, thus abstracts in meetings and conferences were not included in our study. The PICOS principle was used to establish the inclusion and exclusion criteria:

(1) subjects in JIA groups should be diagnosed as JIA according to well-established criteria such as American College of Rheumatology criteria,^[18]^ ILAR classification ^[19]^ or Durban criteria;^[20]^
(2) subjects enrolled in control groups should be healthy individuals without a history of JIA and other autoimmune diseases;
(3) the type of included studies was required to be observational studies (case–control or cohort studies) that featured an assessment of the association between TNF-α polymorphisms and risk of JIA;
(4) the association between TNF-α SNP and JIA was expressed as OR and 95% confidence interval (95% CI), consequently all included studies should report the OR along with the 95% CI, or the OR and 95% CI could be calculated based upon the genotype frequencies. In the cases in which several studies reported an overlapping population, the most complete 1 was deemed eligible for inclusion.

### 2.3 Data extraction

Two independent reviewers (X Li and C Liang) did the literature search separately and were blinded to the results of the other investigator. A stringent screening according to the predetermined eligibility criteria was performed to determine potentially eligible article, the results of the literature search were compared afterwards. Data was extracted independently by 2 reviewers from these eligible articles using a standardized data collection sheet, which included first author, country, year of the publication, ethnicity, sample sizes, genotyping method, diagnostic criteria for JIA, frequencies of A allele in JIA groups and control groups, main results of each included study and HWE of control groups. Any discrepancy between the 2 investigators was resolved through discussion until a consensus was reached. The third review author (Z Lv) was consulted if a consensus could not be reached.

### 2.4 Quality assessment

The Newcastle–Ottawa Scale (NOS) for the assessment of non-randomized studies was adopted to assess the risk of bias in case–control studies and cohort studies.^[21]^ Three broad perspectives including selection of cases and controls, comparability of the groups and ascertainment of outcome of interest were assessed using the Star system (http://www.ohri.ca/programs/clinical_epidemiology/oxford.asp). Two reviewers (X Li and C Liang) independently evaluated the methodological quality of included studies. The results of risk of bias assessment were compared afterwards. In cases of discrepancies between 2 reviewers, the third reviewer (Z Lv) was consulted.

### 2.5 Statistical analysis

To test the association between TNF-α 238A/G or TNF-α 308A/G and JIA, the allelic effect of A (the variant allele) versus G (the common allele) was expressed as OR and 95% CI using the genotype frequency data derived from the included studies. Heterogeneity among included studies was estimated using a *Q* test and the Higgins I^2^ test, where *P* > .1 and I^2^ < 50% indicate acceptable heterogeneity. I^2^ values of 0%, 25%, 50%, and 75% were defined as no, low, moderate and high heterogeneity, respectively.^[22]^ We combined the OR of each study using the fixed-effect model if there was no high between-study heterogeneity across the eligible comparisons. Otherwise, a random-effect model was employed.^[23,24]^

As indicated by previous meta-analysis, there was a strong ethnicity-specific effect on the association between TNF-α polymorphisms and JIA risk, thus we performed subgroup analysis by ethnicity. The leave-1-out sensitivity analysis was conducted by removing each included study at a time and reevaluating the resulting effect on pooled results. Begg rank correlation test and Egger linear regression test using Stata version 12.0 (Stata Corp LP) were combined to assess the publication bias if the number of included studies were larger than 5.^[25]^ Publication bias was considered present with *P* < .05. The forest plots and funnel plots were generated via RevMan 5.3 software (Copenhagen: The Nordic Cochrane Centre, The Cochrane Collaboration, 2014).

## 3 Results

### 3.1 Literature search

**Figure 1:**
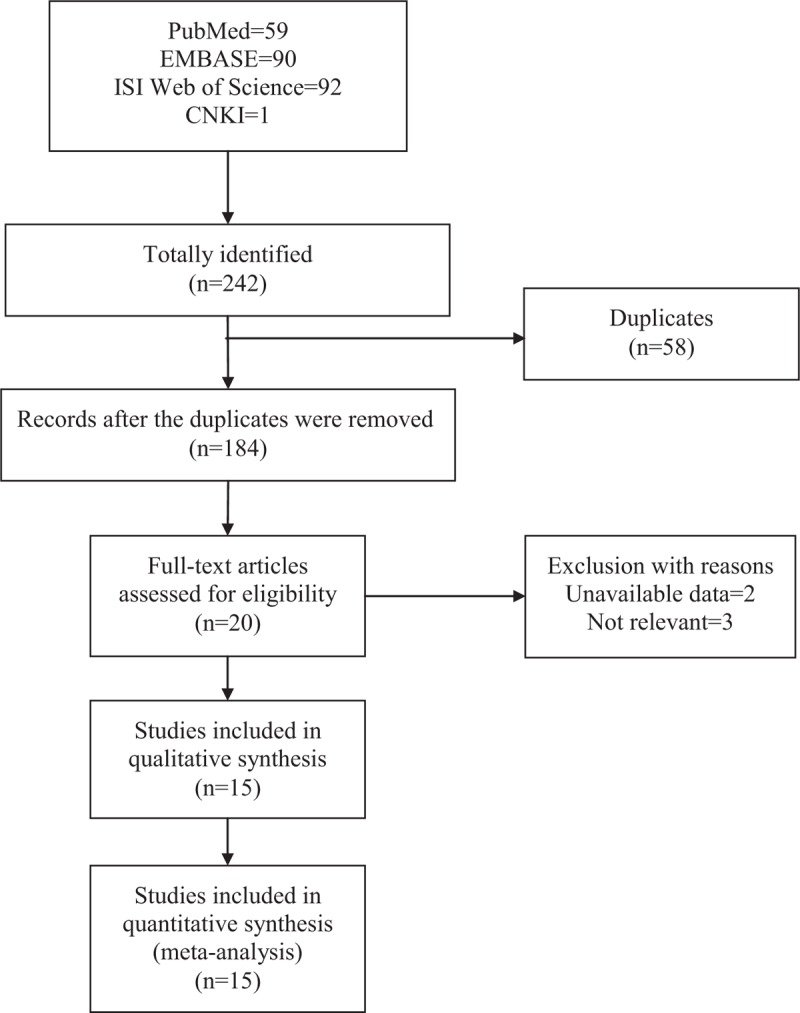
Flow diagram of literature search process.

A total of 200 and 42 studies were identified through literature searches including 59 from PubMed, 90 from EMBASE, 92 from ISI Web of Science, and 1from CNKI (Fig. 1). One hundred eighty-four records were identified after the removal of 58 duplicates for titles and abstracts screen. One hundred sixty-four records were removed because of irrelevance, the remaining 20 records were screened through a full-page browse, and 2 records were deleted for its unavailable data, 3 records were removed because they were not relative to the association between SNPs of TNF and JIA. Finally, 15 studies ^[26–40]^ were included in our meta-analysis according to our strict inclusion criteria.

### 3.2 Main characteristics of included studies

A total of 15 case-control studies published from 2002 to 2016 were identified, including 2845 patients in JIA groups and 4771 patients in control groups. These studies focused on different populations, 12 studies ^[26–34,36,38,39]^ were conducted in Caucasian populations, and the remaining 4 studies were performed in Han,^[40]^ Iranian,^[35]^ Mexican ^[37]^ and Turkish populations ^[31]^ respectively. The sample size of identified records varied from 114 to 1958. All JIA patients were diagnosed according to well-established diagnostic criteria including American College of Rheumatology (ACR) criteria, International League of Associations for Rheumatology (ILAR) classification criteria or Durban classification criteria. The majority of included studies failed to report the HWE of the genotypes distribution of SNP TNF-α-238A/G and TNF-α-308A/G in control groups. The main characteristics and genotypes distribution of TNF-α polymorphisms of included studies were listed in Tables 1 and 2.

**Table 1:**
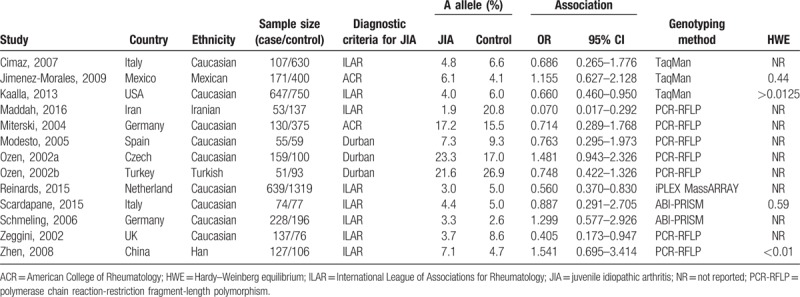
Main characteristics of included studies about the association between TNF-alpha-238A allele and JIA.

**Table 2:**
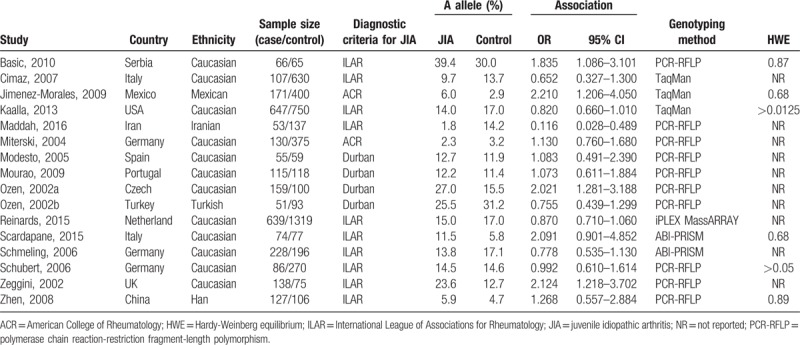
Main characteristics of included studies about the association between TNF-alpha-308A allele and JIA.

### 3.3 Quality of included studies

The NOS was used to assess the methodological quality of included case control studies and the results were summarized in Table 3. Our included studies achieved an average of 5.9 stars, according to the definition and explanation given in each column of the NOS.

**Table 3:**
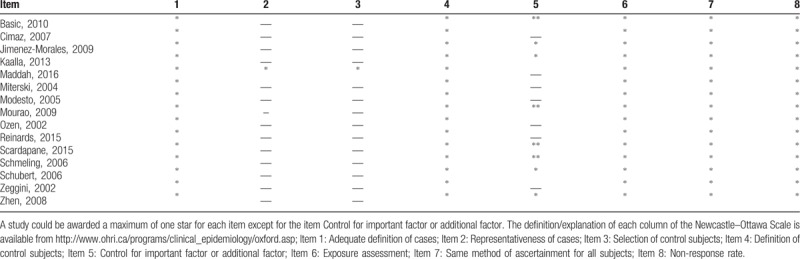
Quality assessment of included case-control studies.

### 3.4 Meta-analyses results

**Figure 2:**
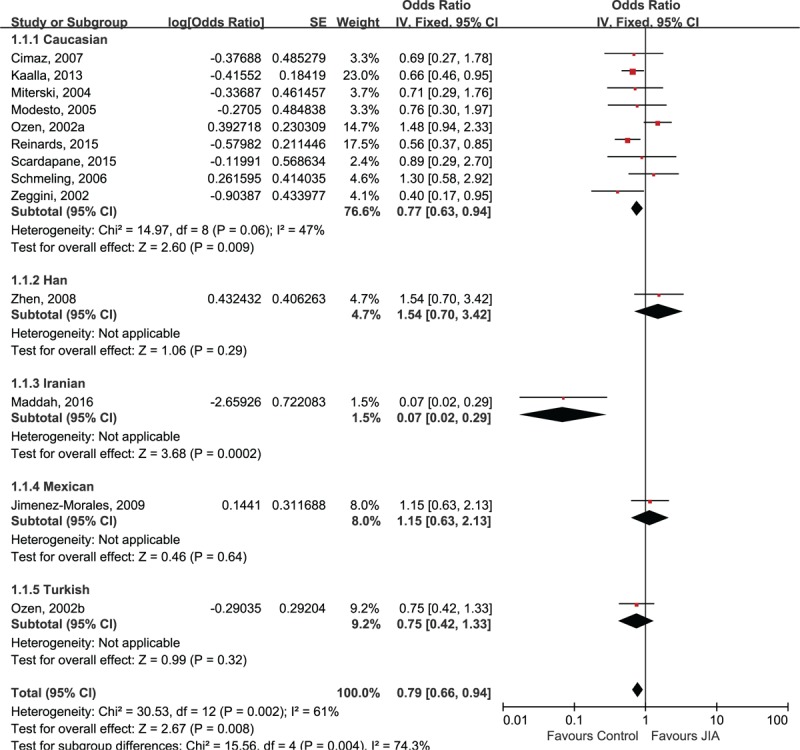
Forest plot of A allele of TNF-α 238A/G and risk of juvenile idiopathic arthritis.

The association between A allele of TNF-α-238A/G and risk of JIA was presented in Figure 2. The pooled OR was calculated for the JIA patients in different ethnic subgroups. There is significant association between the A allele of the TNF-α 238A/G polymorphism and the decreased JIA risk in Caucasian patients (OR 0.77, 95% CI 0.63, 0.94; *P* = .009). The study in Iranian showed similar results (OR 0.07, 95% CI 0.02, 0.29; *P* = .0002) whereas the studies in other ethnicities failed to replicate this finding: Han (OR 1.54, 95% CI 0.70, 3.42; *P* = .29), Mexican (OR 1.15, 95% CI 0.63, 2.13; *P* = .64), and Turkish populations (OR 0.75, 95% CI 0.42, 1.33; *P* = .32).

**Figure 3:**
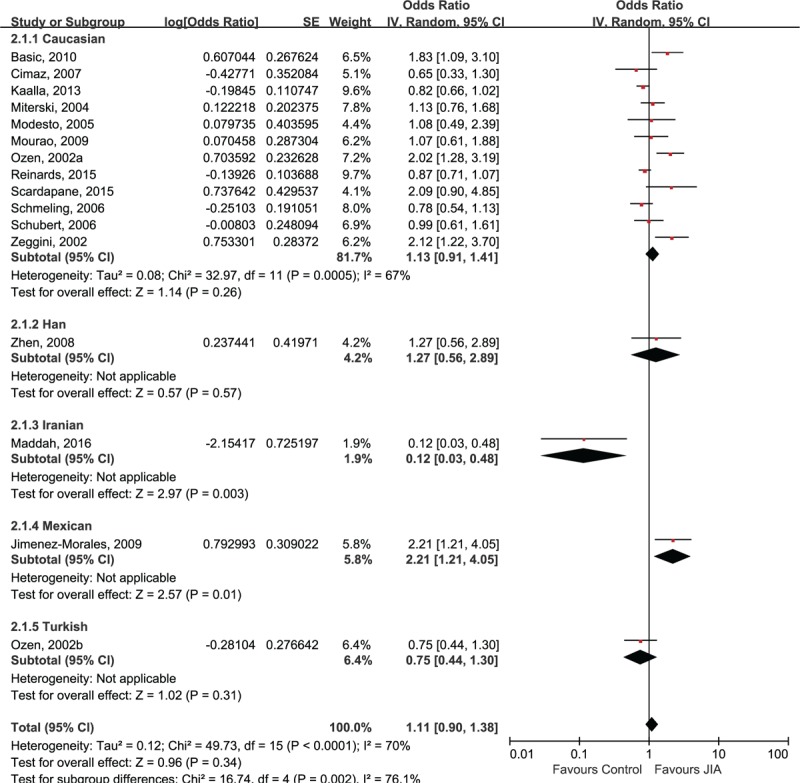
Forest plot of A allele of TNF-α 308A/G and risk of juvenile idiopathic arthritis.

The polymorphism of TNF-α 308A/G was found not to be related to the risk of JIA in all populations (OR 1.11, 95% CI 0.90, 1.38; *P* = .34). When stratified by ethnicity, the pooled ORs in Caucasian populations revealed that A allele of TNF-α 308A/G was not associated with risk of JIA (OR 1.13, 95% CI 0.91, 1.41; *P* = .26). Only the Iranian study indicated a statistically significant association between A allele of TNF-α 308A/G polymorphism and decreased risk of JIA (OR 0.12, 95% CI 0.03, 0.48; *P* = .003), whereas the Mexican population showed a significant association between the A allele of TNF-α 308A/G polymorphism and increased risk of JIA (OR 2.21, 95% CI 1.21, 4.05; *P* = .01)(Fig. 3).

### 3.5 Sensitivity analysis and publication bias

**Figure 4:**
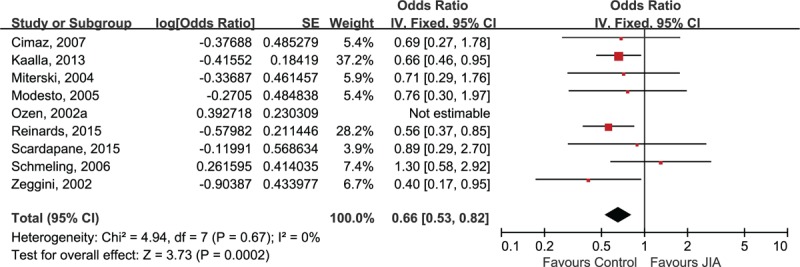
Sensitivity analysis for TNF-α 238A/G and risk of juvenile idiopathic arthritis in Caucasians.

**Figure 5:**
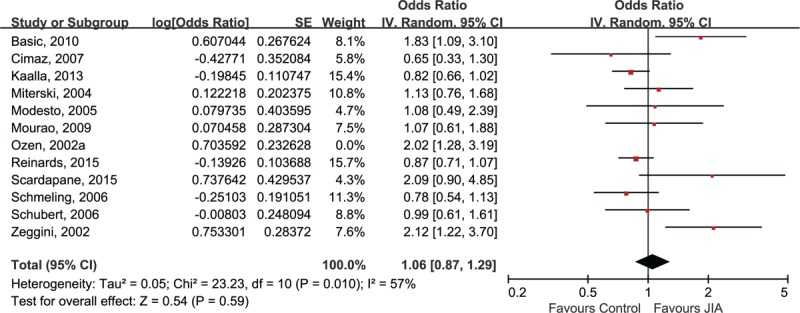
Sensitivity analysis for TNF-α 308A/G and risk of juvenile idiopathic arthritis in Caucasians.

The sensitivity analysis was only performed in Caucasian subjects due to the limited number of studies in other ethnicities. The association between A allele of TNF-α 238A/G polymorphism and JIA remained statistically significant after the removal of any included study (data not shown). However, the heterogeneity across studies was absent after the removal of Ozens’ study (I^2^ = 0%).^[31]^ In comparison to other included studies, Ozen et al reported a much higher A allele frequency in both JIA group (Ozen: 23.3% vs mean: 8.3%) and control groups (Ozen: 17.0% vs 10.2%). As a result, Ozens’ study was excluded in our meta-analysis (Fig. 4). The sensitivity analysis also indicated that the association between TNF-α 308A/G was stable because the negative finding remained unchanged after the exclusion of each study. Since the Ozens’ study reported much higher A allele frequency in their study, we excluded this study in our repeated meta-analysis, resulting in a decline of heterogeneity declined to I^2^ = 57% (Fig. 5).

**Figure 6:**
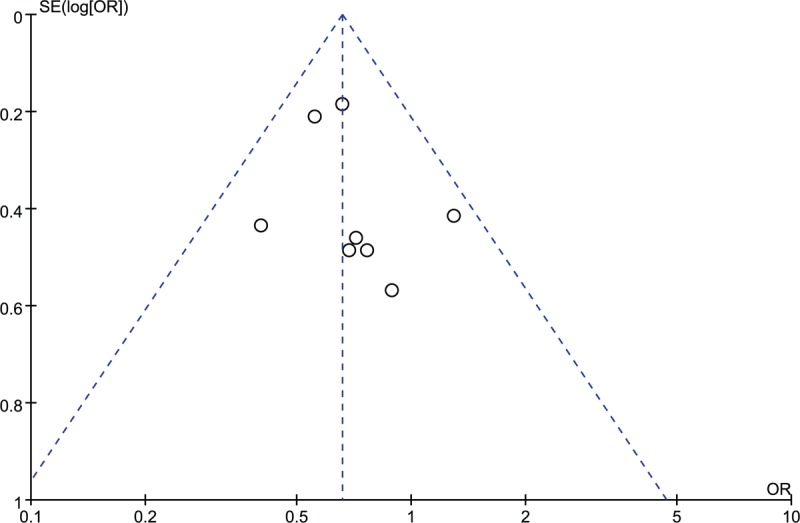
Funnel plot of TNF-α 238A/G and risk of juvenile idiopathic arthritis in Caucasians.

**Figure 7:**
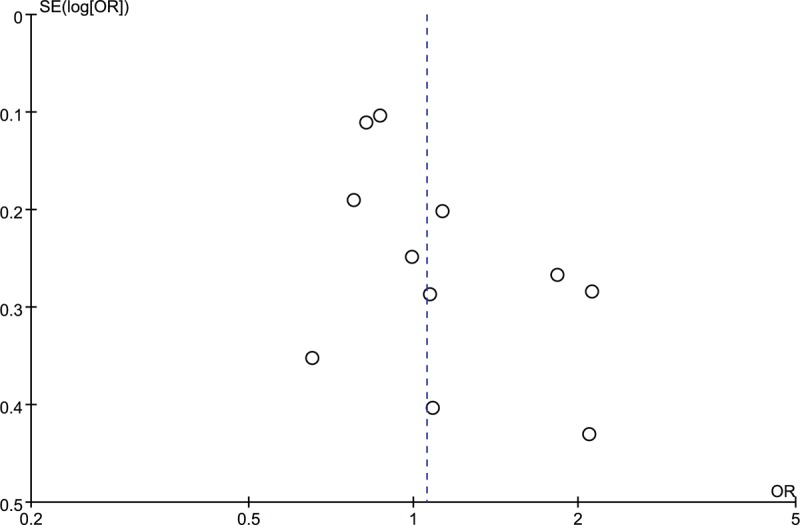
Funnel plot of TNF-α 308A/G and risk of juvenile idiopathic arthritis in Caucasians.

The funnel plots were symmetric and thus suggesting no obvious publication bias (Figs. 6 and 7). The Begg's test (TNF-α 238A/G: z = 0.87, *P* = .386; TNF-α 308A/G: z = 1.40, *P* = .161) and Egger test (TNF-α 238A/G: t = 0.88, *P* = 0.415; TNF-α 308A/G: t = 2.20, *P* = .056) also reveal no statistically significant publication bias.

## 4 Discussion

In our meta-analysis, a total of 15 studies (2845 patients in JIA groups and 4771 patients in control groups) were identified and included in our present study, which were composed of 5 ethnicities from 12 countries. Meanwhile, the stratification by ethnicity was performed to confirm the ethnicity-dependent effect on the relationship between the SNP of TNF-α and the risk of JIA. The results of the meta-analyses indicated that there was a significant association between the A allele of the TNF-α 238A/G but not TNF-α 308A/G polymorphism and a decreased JIA risk in Caucasian patients. The pooled ORs for A allele of the TNF-α 238A/G polymorphism indicated that it conferred a protective role against JIA in Caucasian groups. In terms of polymorphism of TNF-α 308A/G, no significant association for the risk of JIA was found in Caucasians. However, significant findings in the Mexican populations and Iranian were hampered by the limited number of included studies.

A previous meta-analysis for the association between TNF polymorphisms and JIA has been published, which showed no significant relationship between either TNF-α 308A/G or TNF-α 238A/G polymorphisms and JIA in European population.^[16]^ However, the sample size of that meta-analysis was not large enough (7 studies for TNF-α 238A/G and 9 studies for TNF-α 308A/G), and they included an article that did not fit the inclusion criteria for the diagnosis of JIA. In Hohlers’ study, subjects in JIA and control groups were all adults and the “juvenile onset” was defined as onset before age of 40 in their study.^[41]^ In our study, we incorporated sufficient European samples assessing the association between TNF-α 238A/G or TNF-α 308A/G polymorphisms and JIA with the consideration of between-study heterogeneity. But our results did not support the findings of the previous meta-analysis, a statistically significant association between TNF-α 238A/G and decreased risk of JIA was found in Caucasian subjects (OR 0.66, 95% CI 0.53, 0.82; *P* = .0002). We then tried to determine whether the exclusion of Hohlers’ study and the addition of newly identified studies led to the positive association between TNF-α 238A/G and JIA. Based on the forest plot of previous meta-analysis (combined OR 1.25, 95% CI 0.60, 2.57; *P* = .56), the conclusion remained statistically non-significant even after the removal of Hohlers’ study (OR 1.08, 95% CI 0.77, 1.50; *P* = .67), suggesting that the inclusion of more additional studies contributed to the positive finding in our present study. The positive finding was further confirmed by sensitivity analysis and statistically low between-study heterogeneity (I^2^ = 0%). In terms of the association between TNF-α 308A/G and JIA, despite the heterogeneity across studies of the European sample was up to 67%, the stability and reliability of conclusion was validated by sensitivity analysis.

TNF-α, a gene located in the HLA class 3 region, plays an essential role in the pathology process of inflammation and autoimmune disorders.^[8,42]^ A large number of literatures have demonstrated the fluctuating TNF-α concentration in both the plasma and synovial fluid of juvenile with chronic arthritis.^[43–45]^ The fluctuation in levels of TNF-α would affect the levels of multiple cytokines, including IL-1β, IL-6, IL-8, MCP-1, GM-CSF, and CCL5 ^[46]^ as well as several matrix metalloproteinase.^[47]^ These cytokines were further involved in the progression of joint destruction in JIA. Miyamaet et al^[48]^ reported that IL-6 suppressed early differentiation of ATDC5 chondrocyte, which may result in the growth impairment of systemic JIA patients. Meanwhile, MacRae VE and his team demonstrated that the multiple suppressive effects would be exerted on chondrogenic progenitor cells when they were exposed to IL-1β, including the retardation of chondrocyte proliferation and secretion of Aggrecan and Collagen types II and X.^[49]^ TNF-α can also promote the apoptosis of the chondrocyte population and restrain the formation of cartilaginous nodule and the secretion of cartilage-specific proteoglycan.^[50,51]^ It has been shown that TNF-α regulated the cartilage metabolism by complicated signaling pathways, involving the induction of IGF-1 resistance, suppressor of cytokine signaling proteins (SOCS)-3, activation of the intracellular tyrosine kinase JAK2, as well as several cellular proteins.^[52–55]^ For the promotor of TNF-α, several polymorphisms have been studied to explore whether there was any relationship between the polymorphisms and the function of TNF-α.^[56,57]^ Among the polymorphisms, 2 of the most important were SNP TNF-α 238A/G and TNF-α 308A/G, which are associated with enhanced TNF-α production and TNF- α secretion.^[58,59]^ And it has been demonstrated that the TNF-238 polymorphism could induce the CpG methylation site to regulate the gene transcriptional rate.^[60]^

Zeggini and coworkers for the first time identified 4 haplotypes (−1031, −863, −308, +489) that could be associated with risk of JIA.^[26]^ Haplotype-tagging SNPs were determined for transmitted and non-transmitted haplotype groups separately since findings of non-transmitted haplotypes could represent SNP patterns observed in the normal population whereas data derived from the tests of transmitted haplotypes reflect findings in patients with JIA. Maddah et al ^[35]^ observed a significant positive association for TNF-α GG haplotypes (positions −308, −238) in patients with JIA compared to healthy volunteers (*P* < .01), a notable negative haplotypic association was found for TNF-α AG and GA haplotypes at the same positions in case group when comparing with control group. However, only 2 of our included studies evaluated the association between TNF-α polymorphisms and risk of JIA at the haplotypic level, definite conclusion could not be drawn owing to limited number of included studies.

Several potential limitations of this meta-analysis should be acknowledged. First and the most important is the insufficient number of sample sizes. Though the sample size of European is plenty, the number of other ethnicity is not large enough, which may lead to the inaccurate assessment for the association between TNF-α polymorphisms and susceptibility to JIA in other ethnic backgrounds. Although we found a protective effect of the A allele of TNF-α 238A/G against the occurrence of JIA, this conclusion could not be generalized amongst other ethnicities. Secondly, the disease categories of JIA were classified into 7 subtypes according to the classification of ILAR.^[19]^ However, a meta-analysis based on JIA subtype could not be conducted because of the limited quantity of literature. Third, despite of complex genetic diseases, the phenotype and organ specificity of JIA were associate with other confounding factors, involving in age at disease onset, gender, the level of ANA auto-antibodies and so on.^[61,62]^ Therefore, more clinical materials are required to exactly validate the correlation between TNF-α polymorphisms and JIA subtypes.

In conclusion, our study illuminates the association between TNF-α 238A/G and TNF-α 308A/G polymorphisms and JIA in different ethnicities. The findings indicate that the A allele of the TNF-α 238A/G polymorphism would decrease JIA risk in Caucasian subjects, but A allele of TNF-α 308A/G cannot confer a protective role for JIA. The potential role of TNF-α polymorphisms in JIA could not be confirmed in other ethnicities due to insufficiency of available data. Despite potential limitations, we provided a high-quality and reliable result about the role of TNF-α polymorphisms in the occurrence and progression of JIA.

## Author contributions

Zheng-tao Lv produced the idea of this study and was responsible for making the final version of this paper. Xing-yan Li and Chun-hua Liang did the literature search, screened the potentially eligible studies and evaluated the data from each included study, they both contributed equally to this work. Virginia Parkman was involved in revising the manuscript, including some important intellectual content and English editing. Zheng-tao Lv had full access to all of the data in the study and takes responsibility for the integrity of the data and the accuracy of the data analysis.

**Conceptualization:** Zheng-tao Lv.

**Data curation:** Xing-yan Li, Chu-hua Liang, Virginia Parkman, Zheng-tao Lv.

**Formal analysis:** Xing-yan Li, Chu-hua Liang, Zheng-tao Lv.

**Funding acquisition:** Xing-yan Li.

**Investigation:** Xing-yan Li, Chu-hua Liang.

**Methodology:** Xing-yan Li, Chu-hua Liang, Virginia Parkman.

**Resources:** Xing-yan Li, Chu-hua Liang.

**Software:** Xing-yan Li, Chu-hua Liang, Virginia Parkman, Zheng-tao Lv.

**Supervision:** Zheng-tao Lv.

**Validation:** Xing-yan Li, Chu-hua Liang.

**Visualization:** Xing-yan Li, Chu-hua Liang.

**Writing – original draft:** Xing-yan Li, Chu-hua Liang.

**Writing – review & editing:** Xing-yan Li, Chu-hua Liang, Virginia Parkman, Zheng-tao Lv.

Zheng-tao Lv orcid: 0000-0002-8238-7194.
